# Enhancing methane production from food waste fermentate using biochar: the added value of electrochemical testing in pre-selecting the most effective type of biochar

**DOI:** 10.1186/s13068-017-0994-7

**Published:** 2017-12-14

**Authors:** Carolina Cruz Viggi, Serena Simonetti, Enza Palma, Pamela Pagliaccia, Camilla Braguglia, Stefano Fazi, Silvia Baronti, Maria Assunta Navarra, Ida Pettiti, Christin Koch, Falk Harnisch, Federico Aulenta

**Affiliations:** 1 Water Research Institute (IRSA), National Research Council (CNR), via Salaria km 29,300, 00015 Monterotondo, Italy; 2 Institute of Biometeorology (IBIMET), National Research Council (CNR), via G. Caproni 8, 50145 Florence, Italy; 3 Department of Chemistry, Sapienza University of Rome, Piazzale Aldo Moro 5, 000185 Rome, Italy; 4 Department of Environmental Microbiology, Helmholtz-Centre for Environmental Research GmbH—UFZ, Permoserstr. 15, 04318 Leipzig, Germany

**Keywords:** Anaerobic digestion, Biochar, Direct interspecies electron transfer (DIET), Electron-donating capacity (EDC), Food waste, Methane

## Abstract

**Background:**

Recent studies have suggested that addition of electrically conductive biochar particles is an effective strategy to improve the methanogenic conversion of waste organic substrates, by promoting syntrophic associations between acetogenic and methanogenic organisms based on interspecies electron transfer processes. However, the underlying fundamentals of the process are still largely speculative and, therefore, a priori identification, screening, and even design of suitable biochar materials for a given biotechnological process are not yet possible.

**Results:**

Here, three charcoal-like products (i.e., biochars) obtained from the pyrolysis of different lignocellulosic materials, (i.e., wheat bran pellets, coppiced woodlands, and orchard pruning) were tested for their capacity to enhance methane production from a food waste fermentate. In all biochar-supplemented (25 g/L) batch experiments, the complete methanogenic conversion of fermentate volatile fatty acids proceeded at a rate that was up to 5 times higher than that observed in the unamended (or sand-supplemented) controls. Fluorescent in situ hybridization analysis coupled with confocal laser scanning microscopy revealed an intimate association between archaea and bacteria around the biochar particles and provided a clear indication that biochar also shaped the composition of the microbial consortium. Based on the application of a suite of physico-chemical and electrochemical characterization techniques, we demonstrated that the positive effect of biochar is directly related to the electron-donating capacity (EDC) of the material, but is independent of its bulk electrical conductivity and specific surface area. The latter properties were all previously hypothesized to play a major role in the biochar-mediated interspecies electron transfer process in methanogenic consortia.

**Conclusions:**

Collectively, these results of this study suggest that for biochar addition in anaerobic digester operation, the screening and identification of the most suitable biochar material should be based on EDC determination, via simple electrochemical tests.

**Electronic supplementary material:**

The online version of this article (10.1186/s13068-017-0994-7) contains supplementary material, which is available to authorized users.

## Background

Anaerobic treatment by methanogenesis is widely used for the stabilization of municipal wastewater sludges and municipal solid wastes and is increasingly being considered also for the treatment of high-strength industrial wastewaters [1, 2]. The chemistry and microbiology of anaerobic treatment are far more complex than for aerobic systems, with the methanogenic conversion of organic matter critically relying on the establishment of cooperative interactions among microbes belonging to diverse trophic groups, including primary and secondary fermentative bacteria, homoacetogenic bacteria, and methanogenic archaea [3–6].

In particular, the close syntrophic interaction that is established between acetogenic (i.e., acetate-producing) bacteria (also known as syntrophs) and methanogenic archaea is often regarded as the rate-limiting step of methanogenesis, as its stagnation leads to the accumulation of unfavorable metabolites (e.g., propionic and butyric acid) and decay of the entire methanogenic process.

Indeed, catabolic reactions catalyzed by acetogenic bacteria become energetically favorable only when produced reducing equivalents are efficiently scavenged by their syntrophic partners, namely the methanogenic archaea [7]. Typically, this interspecies electron transfer (IET) process is reported to occur via diffusive transport of soluble electron carriers (e.g., hydrogen and formate) from the acetogens to the methanogens, which lie in the proximity one of each other [5, 8]. Low concentrations of electron carriers, however, often result in slow diffusion rates, causing IET to be the bottleneck in the anaerobic treatment process.

Recently, direct interspecies electron transfer (DIET) has been proposed as an alternative strategy to interspecies H_2_/formate transfer, through which microbial species in a community share reducing equivalents to drive the methanogenic degradation of organic substrates [4, 9, 10].

Direct interspecies electron transfer (DIET) can proceed via biological electrical connections or a combination of biological and non-biological electron transfer components.

DIET in syntrophic methanogenic cultures, that is solely based on biological electrical connections, has been documented in co-cultures of *Geobacter* species [11, 12] as well as in co-cultures of *Geobacter metallireducens* with *Methanosaeta harundinacea* [13] or *Methanosarcina barkeri* [14] and in co-cultures of *Geobacter hydrogenophilus* and *Methanosarcina barkeri* [15]. DIET based on biological connections has been documented also in anaerobic granules and methanogenic wastewater digester aggregates [16, 17].

Electrically conductive pili and outer membrane c-type cytochromes have been demonstrated to be important interspecies electron transfer components in *Geobacter* species [9, 18, 19], while the corresponding counterparts of methanogens still remain largely unknown.

Non-biological, electrically conductive materials, including mineral particles and carbon materials, can enhance DIET in syntrophic methanogenic cultures [4], possibly by serving as electron conduits. Many literature studies demonstrated that DIET is stimulated by the presence of (semi)conductive mineral particles [4]. In fact, when micro- and nano-particles of magnetite [20–26] and hematite [22, 26] were added to mixed cultures in the presence of different substrates (acetate, propionate, butyrate, etc.), methanogenesis was significantly accelerated, through a reduction of initial lag phase and enhancement of methane production rates. Recent studies have demonstrated that syntrophic methanogenesis is also promoted by the presence of electrically conductive carbon materials, such as granular activated carbon [14, 15, 27, 28], biochar [24, 29–31], graphite [32], carbon cloth [32] and carbon felt tube electrode [33], that are able to stimulate DIET both in defined co-cultures and in sludge from anaerobic digesters.

So far, the occurrence of DIET has been observed mainly in short-term batch experiments in the presence of single synthetic substrates (such as acetate, propionate, butyrate, etc.…), while its significance, when complex mixtures of real waste substrates are used, has only been marginally examined.

To address this issue, in the present study we investigated the impact and practical viability of biochar supplementation during the anaerobic treatment by methanogenesis of a real waste stream, consisting of a volatile fatty acids (VFA)-rich food waste fermentate (FWF).

Biochar is a carbon-rich solid material produced by the thermal decomposition of diverse biomass species under oxygen-limited conditions [34]. Biochar has been widely considered for carbon sequestration, reduction of greenhouse gas emissions, and as a soil amendment in numerous agricultural and environmental applications [35–38] thanks to its unique properties such as the highly porous structure, alkalinity and high ion-exchange capacity [39]. Due to its porous structure and its relatively high specific surface area, biochar has also been reported to effectively immobilize a variety of environmental contaminants, including metal(loid)s and organic pollutants, subsequently decreasing their bioavailability and ecotoxicity [40–42].

A series of recent studies revealed that biochar has also an apparent role as electron transfer catalyst in redox reactions of biogeochemical and environmental relevance [43]. As an example biochar was found to catalyze the reductive transformation of organic contaminants by facilitating electron transfer from bulk chemical electron donors to the receiving organic compounds [44–46]. Biochar-mediated electron transfer in biological systems has also drawn considerable attention [30–33, 47, 48], serving as a favorable additive material for anaerobic digestion of organic waste without increasing environmental risk.

The surface redox-active moieties (mainly quinone–hydroquinone moieties) of biochar are key components potentially responsible for this electron mediation mechanism, along with the bulk electrical conductivity of the material [49]. However, the underlying fundamentals of the process are still largely speculative and, therefore, a priori identification, screening, and even design of suitable biochar materials for a given biotechnological process is not yet possible [50].

In view of these considerations, the main objectives of this study were (1) to investigate the effect of biochar particles supplementation on methane production from a waste organic substrate, having a relatively complex composition (i.e., the FWF), and (2) to reveal the underlying structure–function properties of different biochars so as to correlate process performance with some specific physico-chemical and electrochemical characteristics of the used materials (i.e., specific surface area, electron accepting/donating capacity; bulk electrical conductivity).

## Methods

### Biochar samples

Three different types of biochar, obtained from pyrolysis of wheat bran pellets, coppiced woodlands, and orchard pruning, were used in this study (Table 1). Specifically, the wheat bran biochar was obtained from wheat bran pellets through a fast pyrolysis process with an average residence time of 3 h at 800 °C in a transportable kiln of 0.8 m in diameter, holding around 30 kg of feedstock. The wood and the orchard biochars were commercial horticultural charcoal (Lakeland Coppice Products, UK) obtained from coppiced woodlands (beech, hazel, oak, birch) and orchard pruning chips (vitis vinifera, pear wood, peach wood), respectively. The wood and orchard biochars were prepared at a pyrolysis temperatures of 500 °C in a transportable ring kiln (215 cm in diameter and holding around 2 tons of feedstock). Prior to being used in the hereafter described batch experiments, the samples were ground and sieved to a size fraction of 1.7–2 mm.

**Table 1:** Properties of biochars used in this study

Acronym	Wheat bran	Wood	Orchard
Original feedstock	Wheat bran pellets	Coppiced woodlands	Orchard pruning
Pyrolysis temperature (°C)	800	500	500
Particle size (mm)	1.7–2	1.7–2	1.7–2
BET specific surface area (m^2^/g)	55 ± 1	61 ± 1	13.7 ± 0.5
Electrical conductivity (S/m)	49.9	1.6	0.5
Total pore volume (cm^3^/g)	0.0445	0.0483	0.0165
Electron donating capacity (meq/g)	0.055 ± 0.01	0.199 ± 0.02	0.298 ± 0.02
Electron accepting capacity (meq/g)	0.434 ± 0.05	0.104 ± 0.01	0.404 ± 0.01

### Batch experiments

Batch experiments were conducted in anaerobic 120 mL serum bottles incubated statically, in the dark, at 20 ± 2 °C. Bottles contained 50 mL of food waste fermentate (FWF), 0.5 mL of sodium bicarbonate (10%, wt/wt), and 1.25 g of the selected biochar, corresponding to a final concentration of 25 g/L.

The FWF was obtained from the mesophilic acidogenic fermentation (in 5-L CSTR reactors operated in fed batch conditions) of food wastes collected from the canteen of the research area “Roma 1” of the Italian National Research Council (CNR). In brief, at the end of the fermentation process, the supernatant of the anaerobic reactor was centrifuged at 15,000 rpm for 10 min, to remove biomass and suspended solids, diluted 1:10 with DI water, and then stored in the freezer at − 20 °C until use. The so-prepared FWF was characterized by a soluble COD of approximately 1.5 gCOD/L, mostly comprising (up to 85%) of C1 to C4 volatile fatty acids (VFA).

Upon preparation, all bottles were sealed with Teflon-faced butyl rubber stoppers, flushed with a 70% N_2_/30% CO_2_ gas mixture and inoculated with 0.2 mL of an anaerobic methanogenic culture from a full-scale mesophilic digester fed with waste activated sludge [corresponding to an initial volatile suspended solids (VSS) concentration of 0.20 g/L].

The experimentation consisted of two successive feeding cycles. During the 1st feeding cycle, four different treatments were setup (each in duplicate): an unamended control (not supplemented with conductive particles) and the treatments containing the three different types of biochar particles (wheat bran biochar, wood biochar and orchard biochar). Once all the bottles had completely converted the initial dose of substrates into methane (1st feeding cycle), they were thoroughly flushed with the 70% N_2_/30% CO_2_ gas mixture in order to remove the produced methane and then were re-spiked with a second dose of FWF (2nd feeding cycle). Prior to the start of this 2nd feeding cycle, the unamended control was split and a further control was set up. This additional control was supplemented with non-conductive, acid washed, silica sand particles (Astralpool Spa, Brescia, Italy) sieved to the same size fraction of 1.7–2 mm as of the biochar particles, with the scope to evaluate whether the methanogenic conversion of supplied substrates could also be facilitated by the addition of particles simply serving as a physical support for biomass growth and aggregation.

Each of the above-described experiments was performed in duplicate, and average values of relevant parameters were reported. During both feeding cycles, control bottles, prepared as above but incubated in the absence of FWF, were also set up in order to assess the impact of conductive particles on the endogenous metabolism of the inoculum. In these controls, 50 mL of anaerobic mineral medium was added to the serum bottles in place of FWF. The mineral medium contained the following components: NH_4_Cl (0.5 g/L), MgCl_2_·6H_2_O (0.1 g/L), K_2_HPO_4_ (0.4 g/L), and CaCl_2_·2H_2_O (0.05 g/L). At the start of the incubation, the pH of all the bottles was around 7.5.

The yield (%) of methane production from VFA contained in the fermentate was calculated as the ratio between the cumulatively produced methane and the cumulatively removed VFA, both expressed in Chemical Oxygen Demand (COD) units.

### Analytical methods

During each feeding cycle, the liquid phase and the headspace of the bottles were regularly sampled with syringes for the determination of individual VFAs and methane, respectively. VFAs (i.e., acetate, propionate, butyrate, isobutyrate, valerate) were analyzed by injecting 1 μL of filtered (0.22 μm porosity) liquid sample, pre-acidified with formic acid (to a final concentration of 0.033 mol/L), into a Perkin-Elmer Auto System gas chromatograph (2 m × 2 mm stainless steel column packed with phase 0.3% Carbowax 20 M, 0.1% H_3_PO_4_, 60/80 Carbopack C support, Supelco, USA; N_2_ carrier gas at 20 mL/min; oven temperature at 120 °C; injector temperature at 200 °C; flame ionization detector (FID) temperature at 200 °C).

Methane was analyzed by injecting 50 μL of headspace sample (removed from the bottles with a gastight Hamilton syringe) into a Perkin-Elmer Auto System gas chromatograph [2 m × 2 mm stainless steel column packed with molecular sieve, Supelco, USA; N_2_ carrier gas at 20 mL/min; oven temperature at 150 °C; injector temperature at 200 °C; thermal conductivity detector (TCD) temperature at 200 °C]. Chemical oxygen demand (COD) and ammonia nitrogen (NH_3_-N) were measured on filtered (0.22 μm porosity) liquid samples with Merck^®^ Spectroquant kits and according to the Nessler method, respectively.

### Physical characterization: determination of surface area and porosity

Surface area was determined according to the Brunauer–Emmett–Teller (BET) multipoint method, through N_2_ adsorption/desorption measurements, carried out at the liquid nitrogen temperature (− 196 °C), using a Micromeritics ASAP 2010 equipment. To this aim, the samples were pre-treated under vacuum at 200 °C for 2 h. The pore distribution was determined from the adsorption isotherms, according to the Barret–Joyner–Halenda (BJH) method [51]. The total pore volume was obtained by the rule of Gurvitsch [52].

### Electrochemical characterization of biochars

#### Electron-accepting and electron-donating capacities

The electron-accepting capacity (EAC) and electron-donating capacity (EDC) of biochars were quantified via mediated electrochemical reduction (MER) and oxidation (MEO) experiments, according to a previously published method [43]. These experiments were carried out in an *H*-type electrochemical cell that was equipped with a 3-electrode setup and was operated at ambient temperature. A glassy carbon rod (Sigradur G, HTW, Germany), a graphite rod (Sigma-Aldrich, Italy) and a KCl-saturated silver/silver chloride electrode (Amel, Italy) were used as the working electrode, counter electrode and reference electrode, respectively. To quantify the EAC and the EDC of biochars, MER and MEO were conducted at an applied potential of − 0.69 and + 0.41 V (vs. KCl-saturated Ag/AgCl), respectively. Diquat dibromide monohydrate (DQ) and 2,2′-azino-bis(3-ethylbenzthiazoline-6-sulfonic-acid) diammonium salt (ABTS) were used as electron shuttles in MER and MEO experiments, respectively. Two types of biochars suspension (4 g/L) in anoxic buffer solution (pH 7, 0.1 M phosphate, 0.1 M KCl) were used in electrochemical tests: (i) sodium borohydride (NaBH_4_) pre-reduced biochar suspensions were employed to determine the EDC; (ii) air-exposed biochar suspensions were employed to determine the EAC.

Briefly, after the WE was equilibrated to the desired redox potential (*E*_h_ = − 0.69 V in MER and + 0.41 V in MEO), 2 mL of stock solutions (11 mM) of the electron transfer mediators DQ (in MER) or ABTS (in MEO) was added to the cells [already containing in each compartment 150 mL of an aqueous solution of KCl (0.1 M) and K_2_HPO_4_ (0.1 M) at pH 7], resulting in reductive and oxidative current peaks, respectively. After re-attainment of constant background currents, different volumes (i.e., 0.5–15 mL) of the biochars suspensions were spiked to the cells. The resulting reductive (MER) and oxidative (MEO) current peaks were integrated to yield the EAC and EDC (both as mmol eq/g biochar) of the biochars added:

1 $${\text{EAC}} = \frac{{\mathop \smallint \nolimits \frac{{I_{\text{rid}} }}{F}{\text{d}}t}}{{m_{\text{char}} }}$$

2 $${\text{EDC}} = \frac{{\mathop \smallint \nolimits \frac{{I_{\text{ox}} }}{F}{\text{d}}t}}{{m_{\text{char}} }}$$

where *I*_red_ and *I*_ox_ (in A) are the baseline-corrected reductive and oxidative currents in MER and MEO, respectively, *F* is the Faraday constant, and *m*_char_ is the mass (in grams) of added biochar.

#### Measurement of the electrical conductivity of biochars

Measurements of electrical conductivity of biochars under compression (in order to minimize contact resistance among particles) were carried out following previously reported protocols [49, 53].

In brief, about 115 mg of biochar powder (< 25 µm) was pressed between two cylindrical steel pistons (diameter 13 mm) applying a weight force of 2.5 tonnes for 30 s. The resulting tablets were placed within a two-electrode cell made up of a Teflon body, ensuring the electrical contact between the biochar and the current collectors. For each type of biochar, the measurement of electrical resistance was performed from the analysis of the voltammetric response, recorded during a potential scan between 0.01 and 0.5 V, at a scan rate of 1 mV/s. The resulting current, flowing between the two electrodes during the potential scan, was recorded and the electrical resistance was calculated according to the Ohm’s law (Eq. 3).

3 $$V = R \cdot I$$

where *V* is the potential (*V*), *R* is the electrical resistance (Ω) and *I* is the current (A).

The electrical conductivity (*σ*) can then be obtained from the electrical resistance (Eq. 4).

4 $$\sigma = \frac{l}{R \cdot S}$$

where *σ* is the electrical conductivity (S/m), *l* is the length (m) and *S* is the section (m^2^) of the tablet.

### Molecular and microscopy analysis of the microbial communities

Fluorescence in situ hybridization (FISH) analysis was performed on paraformaldehyde-fixed samples (2% v/v final concentration, for 24 h at 4 °C), according to a procedure described elsewhere [54, 55]. Oligonucleotide probes specific for Bacteria (EUB338I-III, labeled with fluorescein FLU) and Archaea (ARC915 probe, labeled with indocarbocyanine fluorescent dye, CY3) domains were used. Details of the employed oligonucleotide probes are available at probeBase [56].

To visualize specific cells within the 3D structure of the aggregates, FISH was combined with confocal laser scanning microscopy (CSLM; Olympus FV1000) [54, 57]. The hybridized bacterial cells were excited with the 488 nm line of an Ar laser (excitation) and observed in the green channel from 500 to 530 nm (emission). Archaea cells were excited with the 543 nm line of a He–Ne laser and observed in the red channel from 550 to 660 nm. Silica sand and biochar particles were visualized by their reflection signal (405 nm line of a diodo laser). The three-dimensional reconstruction of CSLM images was elaborated by the software IMARIS 7.6 (Bitplane, Switzerland).

### Chemicals

Redox mediators (i.e., DQ and ABTS) were purchased from Sigma-Aldrich (Milan, Italy) and were used as received. Methane (99.9+%) was purchased from Scott Specialty Gases (Bellefonte, PA). All the other chemicals used to prepare analytical standards or feed solutions were of analytical grade and were used as received.

## Results and discussion

### Influence of biochar particles on the methanogenic degradation of FWF

**Fig. 1:**
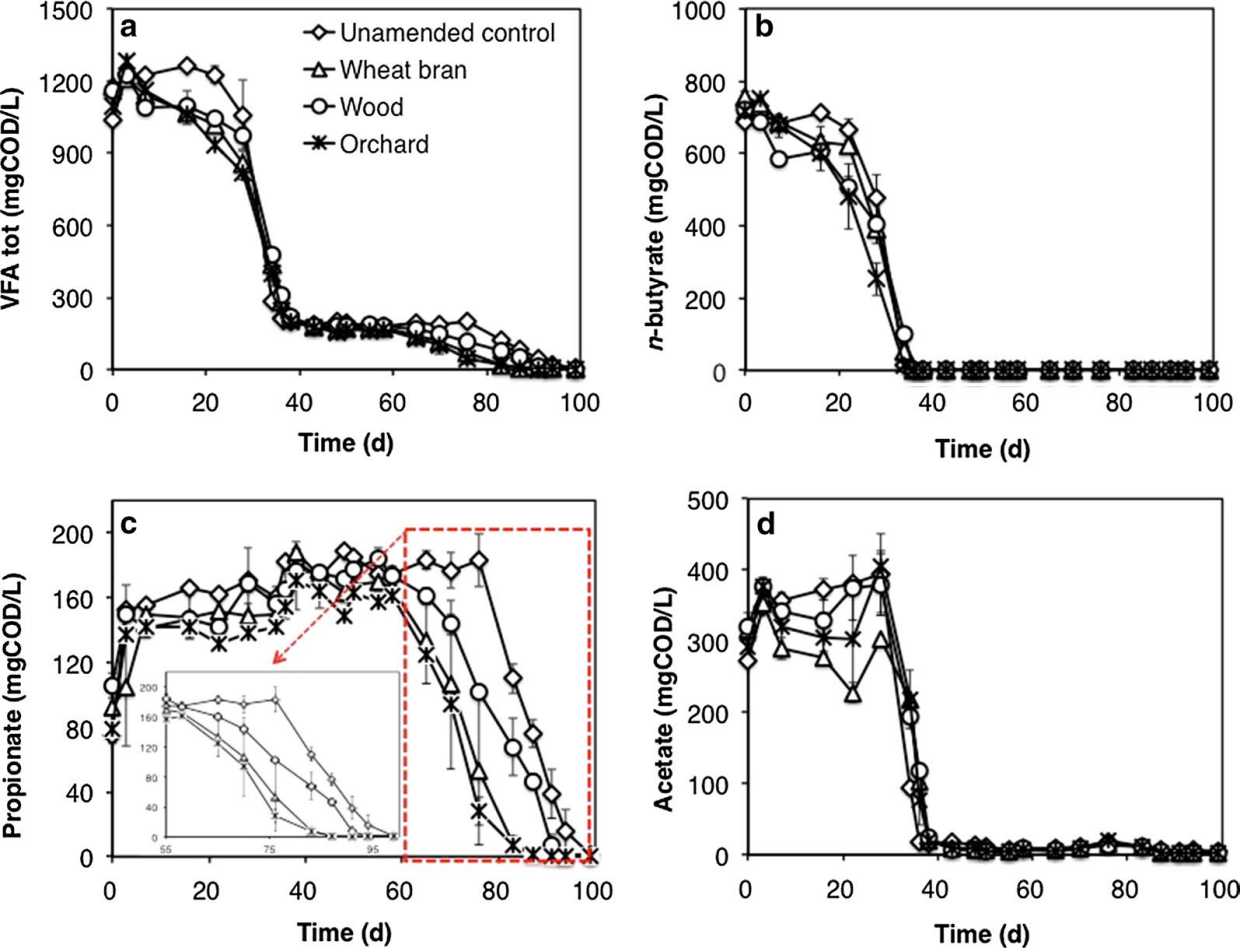
Time course of total VFAs (**a**), *n*-butyrate (**b**), propionate (**c**), and acetate (**d**) concentration in bottles supplemented with conductive particles and in unamended controls, during the 1st feeding cycle. Error bars represent the standard deviation of two replicate bottles

Figure 1a–d shows the time course of VFAs concentration in the different treatments, during the 1st feeding cycle. Interestingly, a statistically significant (*p* < 0.05) effect of biochar was observed only on propionate degradation, which commenced earlier (on day 55 vs. day 76) and proceeded at a higher initial rate in bottles supplemented with conductive particles, relative to the unamended controls (Fig. 1c). By contrast, no statistically relevant differences were observed on acetate and butyrate profiles.

**Fig. 2:**
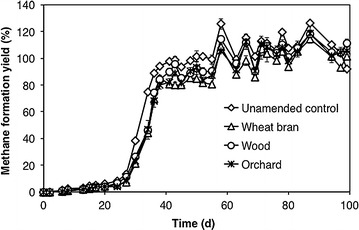
Time course of methane formation yield (%) from fermentate VFAs, in bottles supplemented with conductive particles and in unamended controls, during the 1st feeding cycle. Error bars represent the standard deviation of two replicate bottles

As for methane production, no statistically relevant differences were observed among treatments (Fig. 2), although in all biochar-supplemented bottles methane production (as determined from chromatographic analysis carried out on gas-phase samples) was slightly delayed with respect to the unamended controls, consistently with the reported capacity of biochar to adsorb methane gas [58].

At the end of the 1st feeding cycle (i.e., on day 99), in all bottles methane production accounted for 92–110% of initial VFA (on a COD basis), regardless of the presence of conductive particles (Fig. 2). This finding suggested that methane almost exclusively derived from VFA, while the non-VFA fraction of the COD of the fermentate (approx. 15%) was somewhat recalcitrant to the anaerobic degradation.

**Fig. 3:**
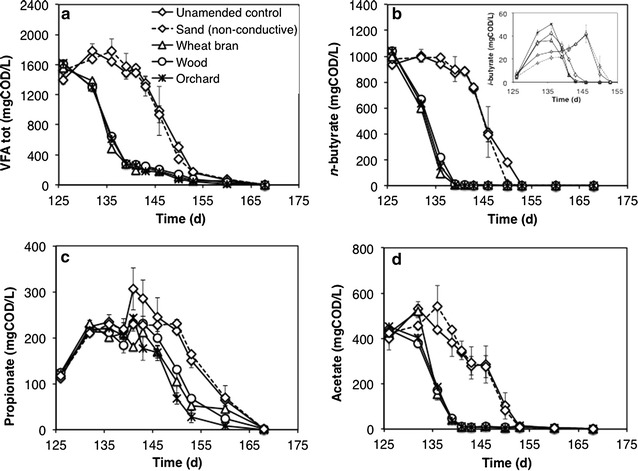
Time course of total VFAs (**a**), *n*-butyrate and *i*-butyrate (**b**), propionate (**c**) and acetate (**d**) concentration (mgCOD/L) in bottles supplemented with conductive particles, with non-conductive sand, and in unamended controls, during the 2nd feeding cycle. Error bars represent the standard deviation of two replicate bottles

On day 126, all bottles were flushed to remove the produced methane and were re-spiked with a 2nd dose of freshly prepared FWF, which could have a slightly different initial composition and concentration with respect to the FWF used during the first feeding cycle. As expected, during this 2nd feeding cycle, in all treatments the overall FWF transformation proceeded at a substantially higher rate compared to the 1st cycle (Fig. 3a–d), most likely due to biomass growth and acclimation on the supplied substrates. Indeed, the complete (91–106%) methanogenic conversion of fermentate VFAs, spiked at an initial concentration of approximately 1500 mgCOD/L, was achieved in less than half of the time needed in the 1st feeding cycle (43 days vs. 99 days).

Notably, in all biochar-amended bottles the lag phase prior to the onset of VFAs degradation was almost eliminated compared to the unamended controls bottles, whereby it typically exceeded 10 days as in the case of butyrate (Fig. 3b).

Importantly, VFAs concentration profiles in control bottles supplemented with non-conductive silica sand (Fig. 3a–d) were almost completely indistinguishable from those of unamended controls, hence providing a further line of evidence that the observed stimulatory effect on methanogenic degradation was ultimately linked to the electrical conductivity of particles, as previously suggested in the literature [9].

**Fig. 4:**
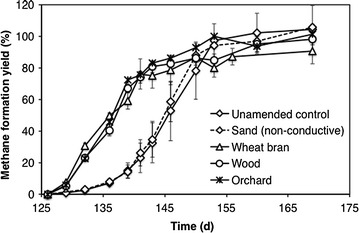
Time course of methane formation yield (%) from fermentate VFAs, in microcosms supplemented with conductive particles, with non-conductive sand, and in unamended controls, during the 2nd feeding cycle. Error bars represent the standard deviation of two replicate microcosms

Differently from the 1st feeding cycle, isobutyrate (insert of Fig. 3b) also transiently accumulated during FWF degradation, reaching a peak concentration of 36–50 mgCOD/L on day 136 in biochar-amended bottles, and on day 146 (of approx. 40 mgCOD/L) in unamended controls and in sand-amended controls (insert in Fig. 3b). In agreement with the observed VFAs time profiles, methane production in biochar-amended treatments proceeded more rapidly to completion with respect to the unamended (or amended with silica sand) controls (Fig. 4). Specifically, the fold enhancement of the initial (from day 129 to day 140) methane production rate in biochar-supplemented bottles relative to the unamended controls was 3.9 ± 0.2 for wheat bran biochar, 4.6 ± 0.2 for the wood biochar, and 5.0 ± 0.1 for the orchard biochar.

### Influence of biochar supplementation on pH and ammonia concentration

Throughout the experimental period, in all treatments the reaction pH remained stable in the range of pH 7.0–7.5, irrespective of the presence of biochar or of non-conductive sand. This finding ruled out the possibility that the observed differences in the methanogenic conversion process could be due to differences in pH values among treatments.

**Fig. 5:**
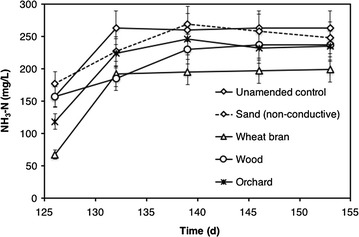
Time course of ammonia nitrogen (mg NH_3_-N/L) concentration in microcosms supplemented with conductive particles, with non-conductive sand, and in unamended controls, during the 2nd feeding cycle. Error bars represent the standard deviation of two replicate microcosms

During to the 2nd feeding cycle, the concentration of ammonia nitrogen (NH_3_-N) was also monitored over time (Fig. 5), in order to evaluate whether the observed improvement in the methanogenic conversion process could be due to the biochar particles alleviating ammonia inhibition via physical adsorption [48]. After an increase during the first 10 days of the 2nd feeding cycle, which was most likely due to the hydrolysis of proteins and/or other ammonia-bearing substrates contained in the FWF, ammonium concentration remained, in all treatments, nearly constant at around 200–250 mg NH_3_-N/L and, hence, values that are substantially lower than those reported to exert inhibitory effects on methanogenic biomass (i.e., > 2 g NH_3_-N/L) [59]. It is also worth mentioning that, throughout the whole experimental period, the observed differences among treatments were not statistically significant, hence providing further indication that the effect of biochar on methanogenic activity was not related to its direct interaction with ammonia.

### Physical and electrochemical characterization of biochars

The three biochars differed markedly for their lumped physical and electrochemical properties (Table 1), which in turn determine their ultimate capacity to interact and/or exchange electrons with soluble (e.g., organic and inorganic substrates) and or insoluble (e.g., microorganisms) components occurring in the surrounding environment.

More specifically, the biochars from wheat bran and wood exhibited a BET-specific surface area (55 ± 1 and 61 ± 1 m^2^/g, respectively) that was substantially higher (i.e., > fourfold) than the orchard biochar (13.7 ± 0.5 m^2^/g). Consistently, wheat bran and wood biochars also displayed total pore volumes that were threefold larger (0.0445 and 0.0483 cm^3^/g, respectively) compared to the orchard biochar (0.0165 cm^3^/g). In all biochar samples, porosity was mostly (40–55%) associated with the presence of micropores having an internal diameter of less than 2 nm, hence unlikely accessible to microorganisms.

Biochar can donate, accept, and/or transfer electrons to/from soluble and insoluble components occurring in its surrounding environment, either via abiotic or biotic pathways [35, 43]. Typically, electron transfer by biochars is reported to involve at least two types of redox-active structures, namely surface-bound quinone/hydroquinone moieties, which are responsible for the electron accepting and donating capacity (i.e., EAC and EDC) of the material, and conjugated *π*-electron systems associated with condensed aromatic sub-structures of the biochars, which are mainly responsible for its bulk electrical conductivity [43].

Here, the EAC and EDC of the different biochars were assessed by means of mediated electrochemical experiments, conducted as reported elsewhere [43]. Further details on the results of mediated electrochemical experiments are included in Additional file 1: Figures S1–S3. As far as the EDC is concerned, the orchard biochar exhibited the highest value (0.30 ± 0.02 meq/g), followed by the wood biochar (0.20 ± 0.02 meq/g), and the wheat bran biochar (0.05 ± 0.01 meq/g). A somewhat similar trend was observed also for the EAC, with the only exception for the wheat bran biochar, which displayed an unexpectedly high EDC value (0.43 ± 0.05 meq/g) and a substantial difference between the EAC and EDC. In spite of that, however, all EAC and EDC values herein determined fall within the range of those reported in the literature for biochars produced under comparable conditions [43].

Interestingly, the trend observed for EDC (with wheat bran exhibiting substantially lower values compared to the wood and orchard biochars) is in agreement with the reported effect of pyrolysis temperature on the chemical composition of the produced biochar. Indeed, the wheat bran biochar was obtained at a pyrolysis temperature of 800 °C, whereas the wood and orchard biochars were at 500 °C. Along this line, it has been reported that EDC (and also EAC) typically increases with pyrolysis temperature up to about 400–500 °C before decreasing at higher temperature values as a consequence of the degradation of previously formed quinoid structures. This latter process is frequently coupled with the onset of aromatization of the biochars that typically starts at around 450–550 °C [35].

The bulk electrical conductivity, a parameter that determines the capability of the material to function as an electron conduit, also differed markedly among the tested biochars, as reported in Table 1. In particular, the measured electrical conductivities of wood and orchard biochars were similar (i.e., 1.6 and 0.5 S/m, respectively), and nearly one order of magnitude lower than that of wheat bran biochar (49.9 S/m), in agreement with the higher pyrolysis temperature at which this latter material was produced (800 °C) relative to the others (500 °C), which possibly resulted in a higher abundance of condensed aromatic and/or graphitic structures.

### Correlating methanogenic activity to the physical and electrochemical properties of biochar

Despite the increasing number of studies proving the capability of biochar and other conductive materials to stimulate the anaerobic digestion process, little efforts have been made, so far, to correlate the extent of the observed stimulatory effect on methanogenic activity to the specific physical and electrochemical properties of the added materials. As a consequence of this lack of knowledge, the choice of the most appropriate materials to be employed remains based on purely empirical considerations, as its impact on the overall process performance can hardly be predicted.

In order to contribute filling this scientific gap, an attempt was made here to correlate the observed performance of the digestion process to the measured biochars properties.

**Fig. 6:**
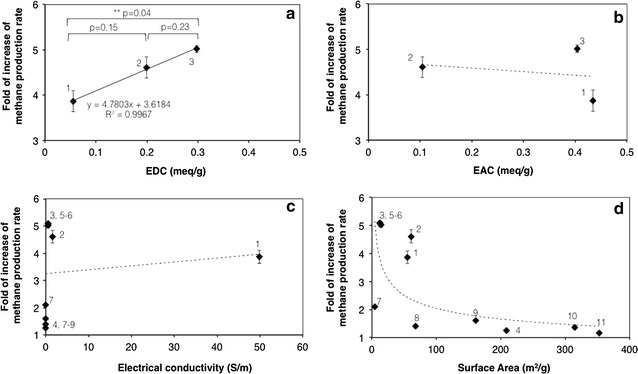
Correlation between the initial methane formation rate (mgCOD/L d) and the electron donating capacity (EDC) (meq/g) (**a**); the electron accepting capacity (EAC) (meq/g) (**b**); the electrical conductivity (S/m) (**c**), and the specific surface area (m^2^/g) (**d**) of the different biochars. Legend: (1) wheat bran (this study); (2) wood (this study); (3) orchard (this study); (4) pine [29]; (5–6) rice straw [62]; (7–9) rice straw [63]; (10) corn stover [64]; (11) pine [64]

To this aim, the fold of increase of the initial methane production rate of the 2nd feeding cycle, relative to the unamended control, was plotted as a function of the EDC, EAC, specific surface area, and electrical conductivity of the different biochars. Interestingly, under the herein applied experimental conditions, a positive linear correlation (*R* ^2^ = 0.9967) was observed exclusively with the EDC (Fig. 6). It should be emphasized, however, that a statistically relevant difference (i.e., *p* < 0.05) among biochars was observed only between the orchard biochar and the wheat bran biochar, whereas no relevant differences were apparent between the orchard and wood biochars and between the wheat bran and wood biochars (Fig. 6a).

To strengthen this analysis, data retrieved from the scientific literature, specifically dealing with the impact of biochar on the methanogenic digestion process, are also included in Fig. 6.

Surprisingly, a lack of correlation was observed between the electrical conductivity and the stimulatory effect on methanogenic activity (Fig. 6c), suggesting that this biochar property was probably over-considered in the scientific literature. Clearly, this finding does not necessarily imply that conductivity is not involved in DIET-driven methanogenic processes [60], rather it suggests that it is not often rate-limiting process performance under most conditions. The fact that, in previous studies, biochar was found to promote DIET with similar rates and stoichiometry as those observed with granular activated carbon (GAC), despite having a nearly 1000-fold lower electrical conductivity with respect to GAC, seems to support this latter hypothesis [31].

As far as the specific surface area is concerned, unexpectedly, data reported in Fig. 6d apparently suggest an inverse correlation between this parameter and the methanogenic activity, with very high surface area biochars being outperformed by low surface area materials. Most probably, this is due to fact that high values of surface area correspond to extremely small pore diameters, often in the range of nanometers, which in turn are too small to be accessible to microorganisms.

It should be noted that according to the best of our knowledge, no other literature studies have examined the impact of the biochar EDC and EAC on the methanogenic degradation process, both suggesting that the herein obtained results will necessarily have to be confirmed in future studies and that EDC and EAC measurements probably deserve greater consideration than previously thought.

### FISH–CLSM analysis

**Fig. 7:**
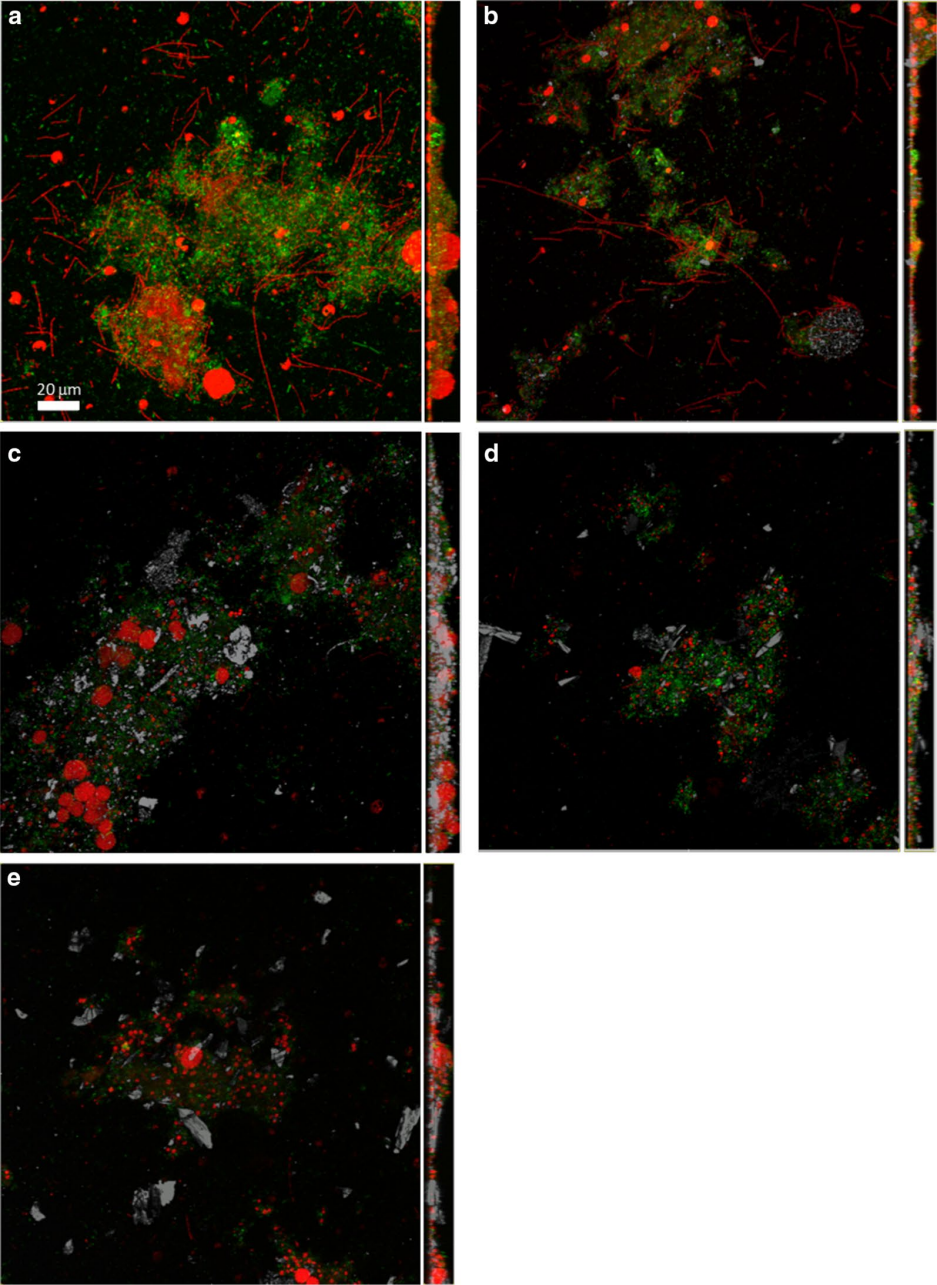
CLSM combined images showing the spatial distribution (*X*–*Y* and *Y*–*Z* planes) of *Archaea* (red) and *Bacteria* (green) cells identified by FISH in aggregates from the unamended control (**a**), silica sand-supplemented control (**b**), wheat bran biochar (**c**), wood biochar (**d**), and orchard biochar (**e**). Biochar particles, visualized by their reflection signal in the same microscopic field, appear gray. Each image is composed by 32–40 optical sections of the aggregate thickness every 0.4–0.5 μm

At the end of the 2nd feeding cycle, suspensions (containing planktonic cells and small biochar and sand particles) from the different treatments were sampled and analyzed by FISH–CLSM in order to visualize the spatial distribution of *Bacteria* and *Archaea* in biochar-supplemented bottles, in unamended controls, and in sand supplemented controls. As expected, irrespective of the treatment, images (Fig. 7) revealed the presence of large microbial aggregates, with archaea (in red) laying in close proximity to bacteria (in green), consistent with the fact that a syntrophic association between these metabolically distinct groups of microorganisms is anyhow necessary during the methanogenic conversion of volatile fatty acids mixtures. Silica sand and biochar particles, visualized by their reflection signal, appear gray (Fig. 7).

The 3D image reconstruction (*x*–*z* plane) clearly showed an intimate association among prokaryotic cells and particles. Interestingly, the aggregate thickness, ranging between 12 and 20 μm, showed the highest values in unamended control (*A*), thus, suggesting that the presence of particles of both conductive and not conductive materials could stimulate bulk biomass compactness.

Confocal microscope analyses of aggregates, in combination with FISH, revealed that the presence of particles also shaped aggregate microarchitecture beyond bulk biomass. Control bottles (Fig. 7a, b) were found to contain a large number of filamentous archaea, resembling the distinct morphology of *Methanosaeta* species, along with irregular spheroid bodies occurring alone or typically in aggregates of cells, resembling the distinct morphology of *Methanosarcina* species. By contrast, in bottles supplemented with conductive materials (Fig. 7c–e), *Methanosaeta*-like filaments were almost completely absent, with *Methanosarcina*-like cells and aggregates accounting for the greatest share of Archaea. Interestingly, previous co-culture investigations clearly pointed out that *Methanosaeta* and *Methanosarcina* species were both capable to participate in DIET, either via direct cell-to-cell electron exchange or via conductive carbon-based materials [9, 13, 14]. Collectively, the results of this study, however, suggest that herein used biochar materials specifically favored the growth of M*ethanosarcina*-like Archaea over *Methanosaeta*-like archaea. This finding is fully in agreement with the results of a previous study showing that *Methanosarcina* preferentially enriched, with respect to *Methanosaeta*, over the surface of coarse biochar particles (2–5 mm) during the methanogenic conversion of glucose [47]. Further microbiological investigations are, however, required to shed light onto this interesting finding.

## Conclusions

This study confirms the capacity of different biochar materials of enhancing the anaerobic methanogenic conversion of food waste fermentate. This is achieved most likely by accelerating rate-limiting interspecies electron transfer (IET) processes (e.g., between acetogens and methanogens), critically involved in the syntrophic conversion of organic substrates, such as volatile fatty acids. Compared with other conductive materials such as iron oxide particles or activated carbon, biochar holds several advantages such as the lower cost (particularly with reference to the activated carbon) and the unique possibility to be sustainably and safely disposed of along with the digestate, thus eliminating the need for further separation and treatment costs. Interestingly, all the herein tested biochars were found to enhance comparably the methanogenic conversion process relative to unamended (or sand-amended) controls, despite being characterized by remarkably different physico-chemical properties (e.g., electron-donating, electron-accepting capacities, surface area, bulk electrical conductivity), with these latter being primarily dependent on the biochar production conditions (e.g., pyrolysis temperature), and the starting lignocellulosic material.

Importantly, for the first time, we could demonstrate that the positive effect of biochar is directly related to the electron-donating capacity (EDC) of the material, yet virtually independent of its bulk electrical conductivity, specific surface area, and electron accepting capacity, properties which were all previously hypothesized to play a major role in the DIET process. Hence, although these results will certainly have to be consolidated through the analysis of the impact of biochars on the anaerobic digestion under a broader range of conditions, they clearly suggest that estimation of EDC via mediated electrochemical tests or hydrodynamic electrochemical techniques with rotating disc electrodes (RDE) [61] may be a straightforward strategy to individuate and select the most effective biochar material and devise possible application strategies.

## Additional file

**Additional file 1.** Additional figures.
