# Supplementary material for: Enhancing methane production from food waste fermentate using biochar: the added value of electrochemical testing in pre-selecting the most effective type of biochar
Source: Biotechnol Biofuels. 2017 Dec 14;10:303. doi: 10.1186/s13068-017-0994-7 (PMC5729428; doi:10.1186/s13068-017-0994-7)

**Additional Material of:**

“Enhancing methane production from food waste fermentate using biochar: the added value of electrochemical testing in pre-selecting the most effective type of biochar”

Carolina Cruz Viggi^1^, Serena Simonetti^1^, Enza Palma^1^, Pamela Pagliaccia^1^,

Camilla Braguglia^1^, Stefano Fazi^1^, Silvia Baronti^4^, Maria Assunta Navarra^2^, Ida Pettiti^2^,

Christin Koch^3^, Falk Harnisch^3^, Federico Aulenta^1,*^

^1^ Water Research Institute (IRSA), National Research Council (CNR), via Salaria km 29,300, 00015 Monterotondo, Italy

^2^ Department of Chemistry, Sapienza University of Rome, Piazzale Aldo Moro 5, 000185, Rome, Italy

^3^ Department of Environmental Microbiology, Helmholtz-Centre for Environmental Research GmbH—UFZ, Permoserstr. 15, 04318 Leipzig, Germany

^4^ Institute of Biometeorology (IBIMET), National Research Council (CNR), via G. Caproni 8, 50145 Firenze, Italy

* Corresponding Author

E-mail: [aulenta@irsa.cnr.it](mailto:aulenta@irsa.cnr.it); Tel: +39-0690672751; Fax: +39-0690672787

**Figure S1.** Reductive (A) and oxidative (B) current responses to increasing amounts of Wheat Bran biochar analyzed by mediated electrochemical reduction (DQ; Eh = −0.69 V vs. Ag/AgCl) and mediated electrochemical oxidation (ABTS; Eh = +0.41 V Ag/AgCl).


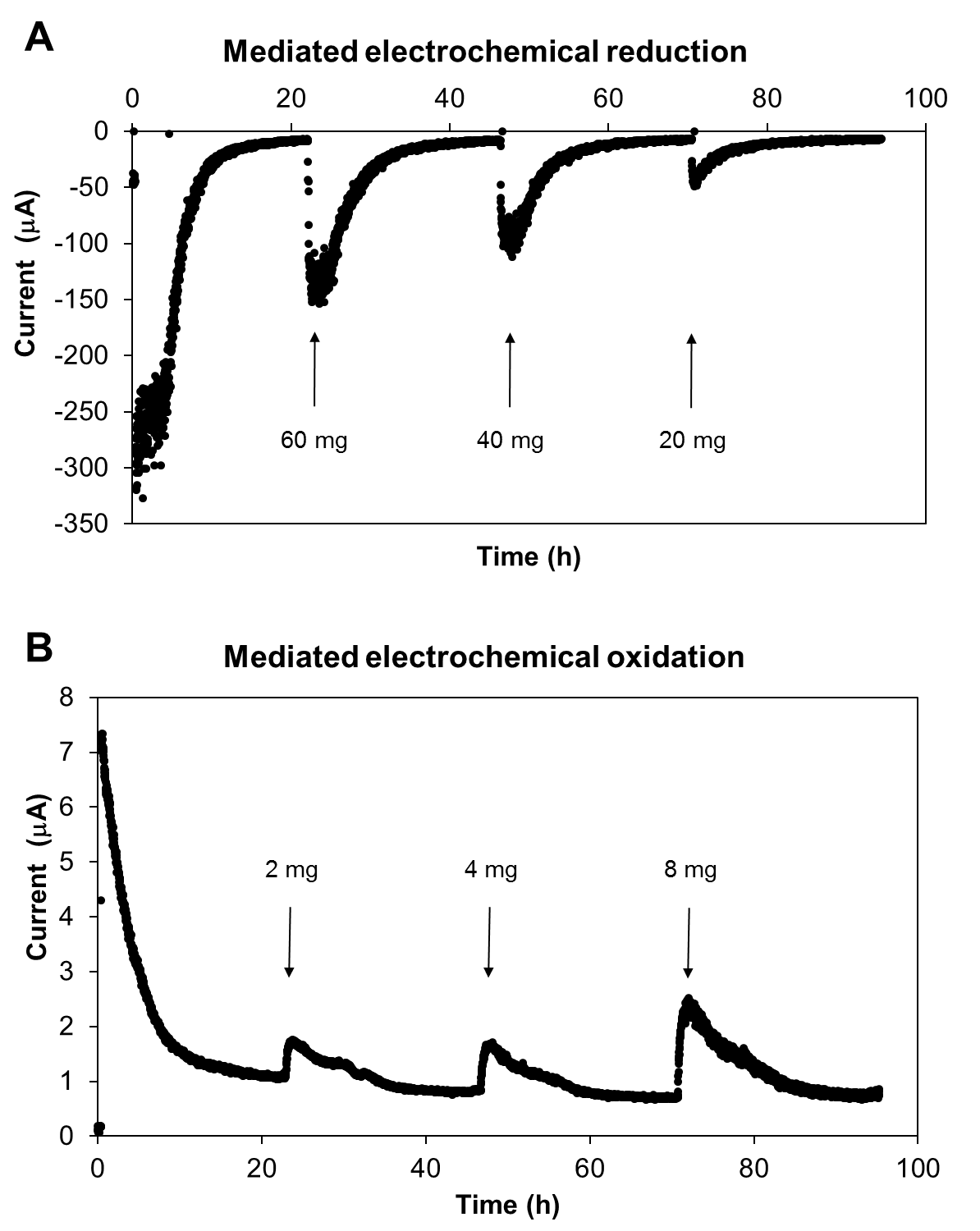


**Figure S2.** Numbers of electrons transferred to biochars (Q) versus biochars added amounts (g). The slopes of the linear regression lines of Q versus added biochar mass correspond to the electron accepting capacity (EAC).


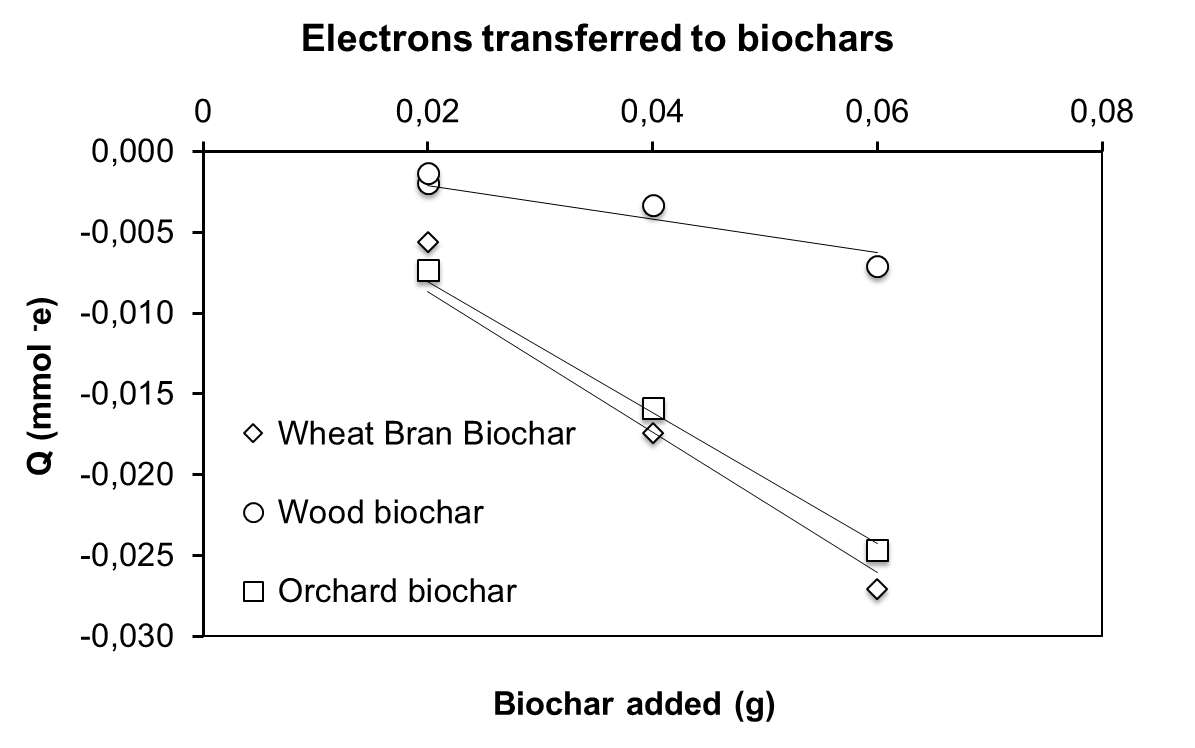


**Figure S3.** Numbers of electrons transferred from biochars (Q) versus biochars added amounts (g). The slopes of the linear regression lines of Q versus added biochar mass correspond to the electron donating capacity (EDC).


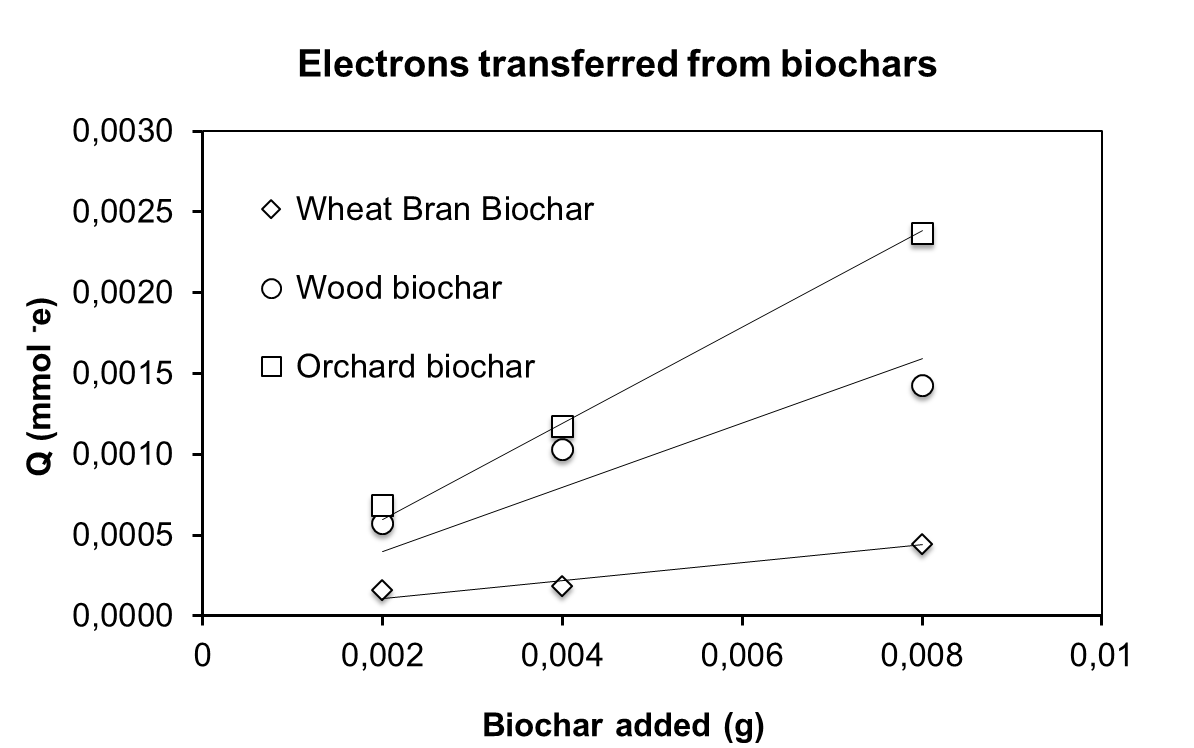

Supplement: Supplementary file 1 — Additional file 1. Additional figures. [file 13068_2017_994_MOESM1_ESM.docx]
